# Taking Fun Seriously: A Scoping Review of Frameworks, Outcomes, and Definitions of Fun in Education

**DOI:** 10.5334/pme.3030

**Published:** 2026-09-18

**Authors:** Stephen K. Stacey, Kurt B. Angstman, Lauren P. Baker, Parivash Badar, Sara S. Oberhelman-Eaton, Rachel S. Wasson, Carol Goulet, Joshua P. Scheil, Heather N. Jett, David A. Cook

**Affiliations:** 1 Department of Family Medicine, Mayo Clinic Health System –Southwest Wisconsin Region, La Crosse, Wisconsin, USA; 2 Department of Family Medicine, Mayo Clinic, Rochester, Minnesota, USA; 3 Department of Psychiatry, Mayo Clinic Health System –Southwest Wisconsin Region, La Crosse, Wisconsin, USA; 4 Department of Psychiatry and Psychology, Mayo Clinic School of Graduate Medical Education, Rochester, Minnesota, USA; 5 Mayo Clinic Libraraies, Mayo Clinic School of Graduate Medical Education, La Crosse, Wisconsin, USA; 6 Division of General Internal Medicine, Mayo Clinic, Rochester, Minnesota, USA

## Abstract

**Introduction:**

Researchers studying the concept of *fun* in education encounter challenges because frameworks, covariates, outcomes, and definitions vary across studies and fields. The authors conducted a scoping review to map the field of fun in education, identify key psychological associations, and synthesize a working definition of fun.

**Methods:**

The authors searched multiple databases in April 2024 for peer-reviewed articles focusing on fun in education and adjacent disciplines. Articles targeting any age group, topic, and educational or workplace setting were included. The authors extracted data on outcomes of fun, conceptual frameworks related to fun, features of fun activities, and definitions related to fun.

**Results:**

Of 1,073 articles screened, 40 were included. The psychological construct of fun was commonly associated with positive emotions experienced in relation to an enjoyable activity. The authors identified 120 distinct features of fun and grouped these into seven categories: safety, novelty, autonomy, playfulness, purpose, immersion, and social connection. Synthesizing findings from all included articles, the authors propose the following definition of fun: “Fun is a positive emotional experience arising from active engagement in enjoyable activities or experiences, often of a playful or amusing nature, that naturally fosters a desire to sustain or revisit the activity.”

**Discussion:**

These findings suggest a theory-informed conceptual framework of fun in learning that may provide a foundation for guiding future research into fun as an educational tool. This synthesized working definition of fun addresses gaps in prior research by incorporating recurring themes that are adaptable across diverse contexts, including health professions education.

## Introduction

Across diverse fields, education increasingly emphasizes learner self-motivation and wellbeing amid growing concerns about burnout and disengagement [1,2]. Contemporary learning environments are often fast-paced, and cognitively demanding. When these demands are perceived as overly effortful or stressful, learners may disengage from the desirable difficulties necessary for effective learning [3,4,5]. Introducing elements of “fun” into the learning process is emerging as one way to promote effortful learning and motivation [6,7,8]. Despite its widespread use in everyday discourse and its intuitive appeal, “fun” remains undertheorized and inconsistently defined in the educational literature. A deeper understanding of fun—including influential factors, key outcomes, and a clear definition—could enable educators and researchers to more intentionally and effectively use and study fun in education. Such applications may be particularly salient for health professions education (HPE), in which the content is vast, time is limited, and stakes are high [9,10].

Although learners and educators routinely describe educational experiences as “fun,” educational research has not developed a consistent conceptualization of what that term represents. If educational research is to understand and study the experiences that learners themselves identify as fun, the construct must first be conceptualized more consistently. While related constructs such as happiness, enjoyment, and intrinsic motivation have been well-explored within psychology and education, “fun” remains undertheorized [11]. Without a clear conceptual framework, it remains difficult to consistently define, operationalize, study, and apply fun in educational settings. This inconsistency also limits the ability to integrate findings across studies, limiting the accumulation of evidence and refinement of the construct.

It may be tempting to infer that lessons learned from constructs such as enjoyment or intrinsic motivation would naturally apply to fun. Existing frameworks such as flow theory [12] and self-determination theory [6] provide valuable insights into motivation and engagement and offer useful lenses through which the experiences learners describe as fun may be understood. Integrating insights from these and other adjacent frameworks may also advance conceptual understanding of fun across educational settings. These frameworks, however, were developed to explain distinct psychological phenomena rather than the construct that learners and educators themselves commonly describe as “fun”. When learners describe why they choose to participate in or persist with an educational activity, they are unlikely to say they experienced “flow” or that their need for autonomy was satisfied. Instead, they are more likely to say “because it was fun”. Because *fun* is the term spontaneously used to describe these educational experiences, educational research should be able to interpret and study what they mean when they describe learning as fun. Greater conceptual clarity may also help identify when fun enhances learning and when it may inadvertently hinder it [13].

Yet existing research on fun in education remains fragmented, lacking a unifying conceptual framework or commonly accepted definition [6,12,14]. This makes it difficult to meaningfully assess the role of fun in learning outcomes, motivation, and instructional design. When fun has been examined, it has frequently been operationalized as a subjective, post-hoc judgment rather than a construct grounded in theory [15]. This focus obscures the mechanisms by which fun may influence learning and limits the ability of researchers to build on one another’s findings [6]. Without greater conceptual clarity, efforts to incorporate fun into pedagogy risk being anecdotal or ineffective.

To address the fragmented and conceptually heterogeneous state of the literature, we performed a scoping review to map concepts, identify recurring themes, and characterize gaps across diverse fields. Our aims are to: (1) map key themes and conceptualizations of fun across disciplines, (2) identify relationships between fun and established educational and psychological frameworks, and (3) synthesize findings into a working definition of fun to inform future theoretical development and empirical testing. Because conceptual understanding often advances through the integration of insights across disciplines, we intentionally synthesized conceptualizations of fun from education as well as other fields where fun has been studied in order to provide a clearer conceptual foundation for future research and educational design in HPE.

## Methods

We performed a scoping review of literature about fun in education by using the established methodological framework outlined by Arksey and O’Malley [16], Levac et al [17], and Cooper et al [18]. We used Covidence [19] to support study procedures at all stages. Our preliminary study-planning searches identified very few studies addressing fun in either health professions education or education more broadly, and we therefore expanded our search to include conceptually-relevant work across diverse disciplines such as workplace learning, organizational psychology, and other related fields where fun has been researched. Although less directly relevant to classic educational settings, we anticipated a broad search would enable a robust, comprehensive conceptual understanding of this construct.

### Article identification

An experienced medical reference librarian and the lead author developed and iteratively refined the search strategy. We compared the articles against authors’ files and cited publications, revised the search terms, and repeated the search 3 times. Search terms included *fun, education*, *students*, *teaching*, *learning*, *happiness*, and *personal satisfaction*. We also used terms such as *enjoyment*, *motivation*, and *engagement* since they are conceptually related to fun. We anticipated that relevant studies might describe the phenomenon of interest using these related terms. We intentionally adopted a sensitive search strategy to maximize retrieval, with relevance determined during article screening rather than the initial search. Using this strategy, we searched Ovid MEDLINE, PubMed, and Scopus. Final search strategies are outlined in Appendix A.

### Article selection

We included peer-reviewed original research studies, review articles, and editorials that evaluated or discussed the psychological construct of fun as an object of study (noun). Articles were excluded if they used *“*fun” only as a descriptor of another activity (adjective) or if the primary focus of the research was a separate concept (e.g., gamification). We included articles targeting any learner group/setting, including grade school, college, professional education, and workplace education. We did not include conference abstracts because we expected that these would not provide sufficient methodological and conceptual detail. Two authors independently screened all titles and abstracts. All articles approved by either reviewer underwent full-text evaluation by the same pair, with conflicts resolved through consensus. As is common with a scoping review on an emerging topic, the operational criteria used for inclusion decisions evolved over the course of the study as our conceptual understanding crystallized. To accommodate this evolution, we erred on the side of inclusion during early stages, and performed a final re-review for inclusion after we had finalized our inclusion definition. The reference lists of included articles were reviewed to identify additional potentially relevant studies. Newly identified articles were added at the article identification phase and screened using the same eligibility criteria.

### Data extraction and charting

The lead author and a second author independently extracted key data from each reviewed article. Pilot extractions were performed to refine the extraction form and promote consistency between reviewers. The standardized extraction form was iteratively revised throughout the study to accommodate emerging themes and insights. Extracted data included article characteristics, main findings, attributes of fun, conceptual frameworks related to fun, potential benefits and negative effects of fun, features of fun activities, definitions of fun, and illustrative quotes. Classifications were continually updated as new themes emerged. All articles were coded using the final extraction form. Conflicts were resolved through consensus.

### Analysis and synthesis

Data describing definitions, conceptual frameworks, attributes of fun, and reported outcomes were extracted verbatim or summarized from each included article. The team comprised a psychologist, psychiatrist, learners, and educators in order to incorporate diverse perspectives into the analysis. Extracted data were initially coded using open coding, with each unique observation retained as an individual code. The research team independently reviewed these codes to identify recurring observations, concepts, and themes. Related codes were iteratively synthesized through axial coding into broader observations and higher-order themes describing conceptual frameworks, attributes of fun, grouped features of activities that were viewed to enable or inhibit fun, and common themes in the definitions of fun. Throughout the analysis, our interdisciplinary team interpreted findings with attention to their relevance for HPE. All authors reviewed and approved the final synthesis.

## Results

**Figure 1:**
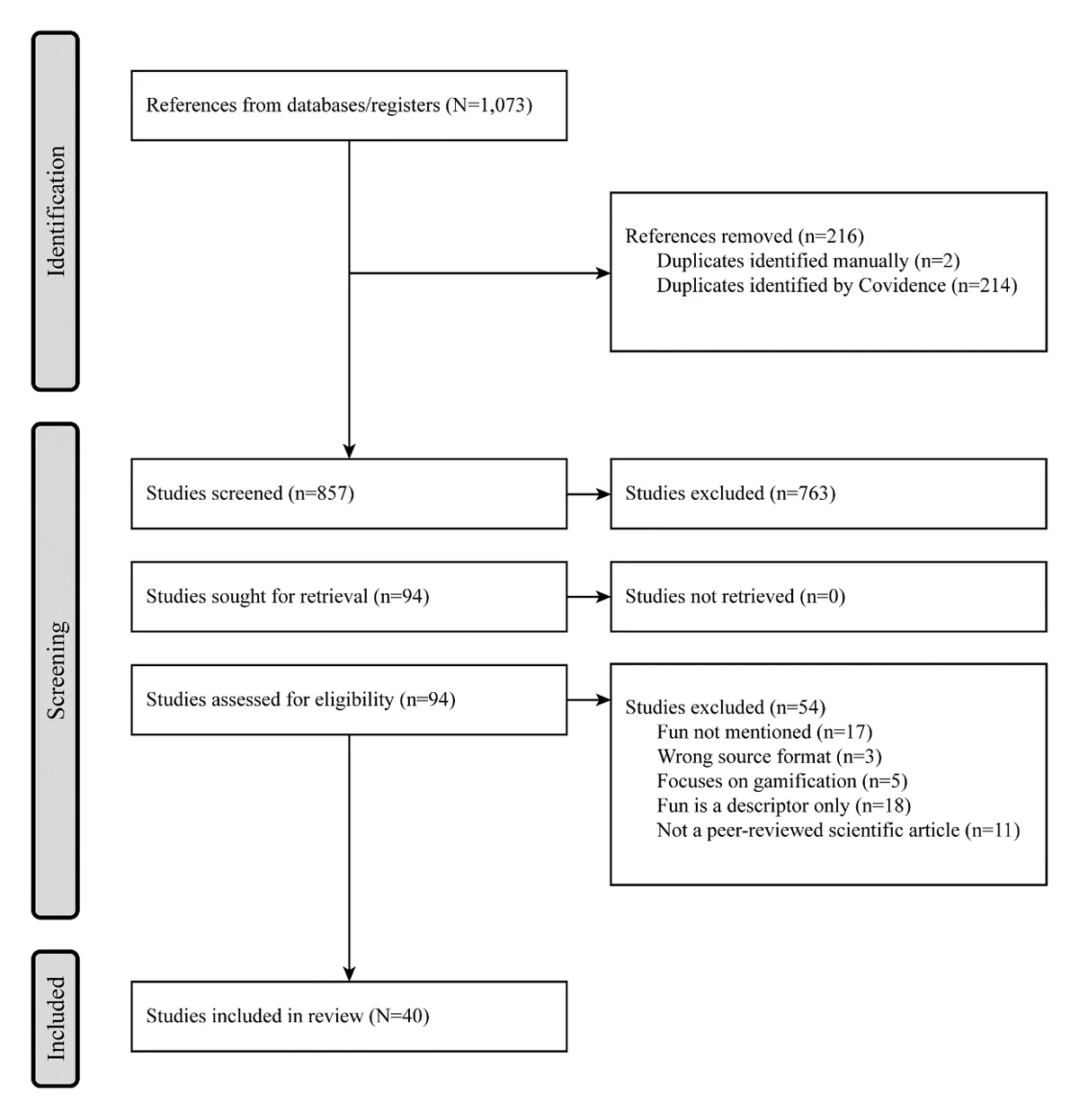
Flow Diagram for a Scoping Review of Fun in Education.

Of 1,073 articles reviewed, we included 40 in our analysis (Figure 1). Key findings are listed in Table 1.

**Table 1:** Key Findings From Articles Identified in a Scoping Review of Fun in Education.


FIRST AUTHOR, YEAR	ARTICLE TYPE	STUDY POPULATION^a^	OUTCOME(S)^b^	FEATURES OF ACTIVITIES^c^		DEFINITION(S)^d^

				ENABLERS	INHIBITORS	

Ainley, 2011	Cross-sectional	Ch, PE		Purp, Imm		0

Baid, 2010	Review/editorial	N/A	Pos (PW, Soc, Motiv, Perf), Neg	Novel, Aut, Play, Imm, Soc	Novel, Imm	0

Becker, 2012	Cross-sectional	Ad, Work, HC	Pos (PW, Soc, Perf), Neg	Play, Purp, Imm, Soc	Aut, Soc	1, borrowed

Bolton, 2009	Review/editorial	N/A	Pos (PW, Soc, Motiv, Perf), Neg	Novel, Aut, Play	Safe, Novel, Aut	0

Chan, 2019	Cross-sectional	Ad, UE	Pos (PW, Soc, Perf)	Novel, Aut, Play, Imm		1, borrowed

Chu, 2017	Qualitative	Ch, PE	Pos (Soc, Motiv, Perf)	Safe, Novel, Aut, Play, Purp, Imm, Soc	Safe, Novel, Aut, Imm, Soc	0

Dismore, 2011	Qualitative	Ch, PE	Pos (PW, Soc), Neg	Novel, Aut, Play, Purp, Imm, Soc	Novel, Aut, Play, Imm, Soc	2, borrowed

Elton-Chalcraft, 2013	Qualitative	Ch, PE	Pos (PW, Motiv), Neg	Safe, Aut, Play, Purp, Imm, Soc	Purp, Imm	1, novel

Fleming, 2005	Qualitative	Ad, Work	Pos (Perf), Neg	Aut, Play, Imm, Soc	Safe, Novel, Aut, Soc	0

Fluegge, 2008	Cross-sectional	Ad, Work	Pos (PW, Soc, Motiv, Perf), Neg	Play, Purp, Soc	Novel, Purp, Soc	3, borrowed, novel, cited

Ford, 2003	Cross-sectional	Ad, Work	Pos (PW, Soc, Motiv, Perf), Neg	Play, Purp, Soc	Safe, Imm	2, novel

Fu, 2009	Test/scale development	Ad, UE	Pos (Soc, Motiv)	Aut, Purp, Imm, Soc		0

Hsbollah, 2022	Qualitative	Ad, UE	Pos (Soc, Motiv, Perf)	Aut, Play, Imm		2, borrowed

Karl, 2005	Cross-sectional	Ad, Work	Pos (PW, Soc, Motiv, Perf), Neg	Novel, Play, Soc	Safe, Aut, Play, Purp, Soc	0

Karl, 2006	Cross-sectional	Ad, Work, HC	Pos (PW, Soc, Motiv, Perf), Neg	Play, Purp	Safe	1, borrowed

Karl, 2007	Cross-sectional	Ad, Edu, Work, HC	Pos (PW, Motiv, Perf), Neg	Imm	Aut, Purp	1, borrowed

Karl, 2010	Experimental	Ad, GE	Pos (PW, Soc, Motiv, Perf), Neg	Play, Purp, Imm	Purp	2, borrowed

Karl, 2006	Cross-sectional	Ad, GE	Pos (PW, Perf), Neg	Play, Purp, Imm		2, borrowed

Kazen, 2015	Experimental	Ad, UE	Pos (Perf)			0

Lamm, 2009	Cross-sectional	Ad, Work	Pos (PW, Soc, Perf), Neg	Safe, Play	Purp, Imm	6, borrowed, novel, cited

Lucardie, 2014	Qualitative	Ad, Edu	Pos (PW, Soc, Motiv, Perf), Neg	Safe, Aut, Play, Purp, Imm, Soc	Safe, Novel, Purp, Imm	0

McDowell, 2004	Test/scale development	Ad, Work	Pos (PW, Soc, Motiv, Perf), Neg	Novel, Aut, Play, Soc	Safe, Novel, Soc	3, novel

Peluchette, 2005	Cross-sectional	Ad, Work, HC	Pos (PW, Soc, Motiv, Perf), Neg	Play, Purp		1, novel

Plester, 2009	Qualitative	Ad, Work	Pos (PW, Soc, Perf), Neg	Safe, Novel, Play, Soc	Safe	1, novel, cited

Pryor, 2010	Review/editorial	N/A	Pos (PW, Soc, Motiv, Perf), Neg	Safe, Aut, Play	Safe, Imm, Soc	2, novel

Read, 2002	Test/scale development	Ch		Imm		0

Reis, 2017	Experimental, qualitative	Ad	Pos (PW, Motiv, Perf), Neg	Soc	Soc	1, novel, cited

Sim, 2006	Test/scale development	Ch, PE	Pos (Perf)			1, borrowed

Strean, 2000	Qualitative	Ch, PE	Pos (Motiv)	Novel, Aut, Play, Purp, Imm, Soc	Novel	3, borrowed, novel, cited

Tasci, 2016	Test/scale development	Ad, Work	Pos (PW, Soc, Motiv, Perf)	Novel, Aut, Play, Imm, Soc	Safe	3, borrowed, novel, cited

Tew, 2012	Experimental	Ad, UE	Pos (PW, Soc, Perf), Neg	Aut, Play, Soc	Imm, Soc	1, borrowed

Tews, 2013	Cross-sectional	Ad, Work	Pos (PW, Soc, Motiv, Perf), Neg	Play, Purp, Imm, Soc		2, borrowed

Tews, 2014	Cross-sectional	Ad, Work	Pos (PW, Soc, Motiv, Perf), Neg	Aut, Soc		3, borrowed

Tews, 2015a	Test/scale development	Ad, UE	Pos (PW, Perf), Neg	Play, Purp, Imm, Soc	Soc	1, novel

Tews, 2015b	Cross-sectional	Ad, Work	Pos (PW, Soc, Motiv, Perf), Neg	Aut, Purp, Imm, Soc		2, borrowed

Tews, 2017	Cross-sectional	Ad, Work	Pos (PW, Soc, Motiv, Perf), Neg	Imm, Soc		1, borrowed

Tews, 2019	Review/editorial	N/A	Pos (Motiv, Perf), Neg	Safe, Aut, Purp, Imm, Soc	Safe, Purp	6, borrowed, novel, cited

Tisza, 2023	Test/scale development	Ch, PE	Pos (PW, Soc, Motiv, Perf)	Aut, Imm, Soc	Safe, Aut	1, borrowed

Vieira, 2017	Review/editorial	N/A	Pos (Soc, Motiv, Perf)	Novel, Play, Imm, Soc		2, novel, cited

Visek, 2015	Qualitative	Ad, Ch, PE	Pos (Soc, Motiv, Perf)	Purp, Soc		0


^a^ Study population: Ad, adults; Ch, children/minors; Edu, educational environment other than PE, UE, or GE; Work, workplace environment/employees; GE, graduate education; HC, health care/medical environment; N/A, not applicable (the article is not a study); PE, primary education (K-12); UE, undergraduate education. ^b^ Outcome(s) of fun identified in the article: Neg, negative effect(s) of fun identified in the article; Pos, positive effect(s) of fun identified in the article (Motiv, motivation; Perf, performance; PW, personal well-being); Soc, social connection. ^c^ Enabler(s) and/or inhibitor(s) identified in the article: Safe, safety; Novel, novelty; Aut, autonomy; Play, playfulness; Purp, purpose; Imm, immersion; Soc, social connection. ^d^ Definition(s) of fun identified in the article: No., number of definitions identified in the article; borrowed, borrowed definition verbatim or nearly verbatim from another source; cited, cited prior work in creating a novel definition; novel, definition appears novel to that article.

### Article characteristics

Of the 40 articles, 5 (13%) were reviews or editorials. The 35 original research studies included 8 (23%) in K-12 education, 6 (17%) in undergraduate college education, 2 (6%) in postgraduate education, 2 (6%) in other educational settings, 16 (46%) in workplace settings, and 2 (6%) in a setting other than formal education or workplace. Twenty-seven (77%) included adults, and 9 (26%) included children. Appendix B contains a summary of main findings and definitions of fun.

### Attributes of fun

We first considered the attributes of fun itself. Emotional states used to describe or define fun have in common their positive valence on the bivariate core affect model, meaning they are good or pleasant [20]. Fun is, however, distinct from these positive states in that it is not thought of as a single emotion but rather as a range of positive emotions connected to activities. As one report said, “Writers should not assert reflexively that fun and enjoyment are synonymous. For example, one might enjoy a good meal, but would not necessarily say it was a fun dinner” [21]. Positive emotional states frequently associated with fun include delight [6], mirth [11], playfulness [22], humor [15], happiness [11], satisfaction [23], excitement [24], amusement [25], and pleasure [26].

Some concepts, such as creativity, were discussed both as contributors to and results of fun, whereas others, such as enjoyment, were seen both as defining and enabling fun [22,24,25,27]. Fun was associated with the presence of positive affect and the absence of negative affect [28]. Whereas some articles highlighted potential negative consequences of fun, no articles used negative emotional or affective descriptions to characterize the experience of fun. As one author noted, “negative emotions are contraindicative for experiencing fun” [12].

Many articles highlighted the complexity of fun, discussing how fun is perceived differently by each person and is influenced by situational and cultural factors [6,11,12,23,26,29,30]. Fun is “often described as being about the activities that [individuals] participated in” [14]. Fun is “an experience that involves active contact with the environment” [28]. Because each person may perceive different activities as enjoyable, fun is difficult to measure objectively or consistently replicate [31]. For example, one person may find a particular learning activity fun, whereas another may perceive the same activity as boring or intimidating.

Fun was described as “a strong, intrinsic motivating factor” [12]. Articles highlighted that it is difficult to have too much fun, and people tend to continue activities as long as they seem fun [32,33]. Fun was hypothesized to influence motivation in various ways. For example, fun activities may be minimally ego depleting or even restorative of a person’s ability to exert self-control and focus [34]. The expectation of positive outcomes and intrinsic enjoyment can drive people to seek out and participate in fun activities. Fun can trigger and maintain interest over time, fostering lasting engagement, commitment, and a desire to return to fun activities [12,13,15]. More details of the attributes of fun are included in Appendix C.

### Conceptual frameworks used in the study of fun

The reviewed articles interpreted or discussed fun through several existing conceptual frameworks, including expectancy theory, flow, goal orientation, constructivist learning theory, performance orientation, and cognitive load, along with others (Table 2). These conceptual frameworks provide theoretical perspectives for understanding how fun has been conceptualized in relation to well-being [28], motivation [6], and engagement [7] across various contexts. As an example, fun was closely linked in several articles to the concept of *flow*, a state in which a person experiences deep immersion and enjoyment in activities [7,12,28]. Another explanatory framework is *engagement*. According to this perspective, people feel intrinsically motivated and invest effort into those activities they find enjoyable and meaningful [6,35,36]. The *3 stages of interest* framework suggests that fun activities may spark initial interest, sustain engagement, and foster lasting commitment [36]. The person’s social context also has a critical role because environments that promote psychological safety and respect social boundaries contribute to a more enjoyable and inclusive experience of fun [6,37].

**Table 2:** Conceptual Frameworks Identified in a Scoping Review of Fun in Education, 2024.


FRAMEWORK	EXPLANATION	EXAMPLE

Affective events theory [6]	“Transitory or ongoing favorable events tend to elicit positive emotional reactions; whereas unfavorable … events elicit negative emotional reactions” [6].	Fun activities are proposed to enhance overall enjoyment and satisfaction.

Autonomous vs external self-control [34]	Autonomous, motivated self-control is less depleting than self-control for extrinsic reasons.	Fun activities are presumed to be less depleting.

Cognitive load [6]	Cognitive load refers to the capacity of our working memory to handle and process information at any given moment.	Fun is hypothesized to impose “an extraneous cognitive load on learners that impedes cognitive processing, resulting in less learning” [6].

Constructivist learning theory [53]	An educational theory that suggests “humans construct knowledge and meaning from their experiences” [15] rather than passively through instruction.	Fun may promote engagement and enjoyment as learners construct their own understanding through interactive and enjoyable activities.

Core affect model [29]	All affects are explained in terms of a combination of 2 dimensions, hedonic valence (negative-positive) and level of arousal (low-high).	While having fun, people experience a positive-valence emotional state. Fun can be experienced as a low- or high-arousal state.

Culture [37]	“A group has a culture when it has enough shared history to have formed a set of basic assumptions that guide behavior, perceptions, thoughts, and feelings” [37].	Culture mediates what may be considered fun in different social groups.

Dimensions of engagement [35,58]	Dimensions of engagement are behavioral engagement (participation and involvement), emotional engagement (focusing on positive and negative reactions), and cognitive engagement (level of investment).	Engagement on these dimensions is hypothesized to mediate the experience of fun.

Ego depletion [34]	Self-control in a resource-demanding task depletes the limited resources that a person needs to carry out subsequent effortful tasks.	Fun activities may reduce ego depletion and help restore a person’s ability to exert self-control and focus on tasks.

Engagement [6,7,46,59]	“Training engagement can be characterized as the harnessing of trainees’ selves in learning endeavors where they immerse themselves physically, cognitively, and emotionally” [6]. Engagement has also been defined as “psychological investment in and effort directed toward learning, understanding, or mastering the knowledge, skills, or crafts that academic work is intended to promote” [8,60].	Fun activities may enhance engagement, making people more likely to invest psychologically and exert effort toward learning, understanding, or mastering the knowledge and skills intended by the task.

Expectancy theory [51]	People are motivated to act when they expect their actions will be successfully performed (expectancy), leading to desired rewards (instrumentality) that they value (valence).	People may be motivated to seek out and participate in activities they perceive as fun, driven by the expectation of positive outcomes and intrinsic enjoyment.

Flow [6,11,12,22,23,28,29,32,56]	“An optimal experience, where the following characteristics were present: (a) an intrinsically rewarding experience, (b) a loss of reflective self-consciousness, (c) a distorted experience of time, (d) an intense and focused concentration on the present, (e) a merge of actions and awareness, (f) an optimal balance between challenge and skills, (g) a sense of control over the situation, and (h) clear goals and immediate feedback” [12].	Flow relates closely to fun as both concepts involve immersive and enjoyable experiences. Fun was viewed as a natural outcome of achieving a flow state.

Four-phase model of interest development [36,61]	Triggered situational interest, maintained situational interest, emerging (less-developed) individual interest, and well-developed individual interest.	Fun may trigger situational interest and is hypothesized to help maintain interest throughout all 4 phases.

Goal orientation [6]	“A preference to grow and develop one’s competence by acquiring new skills and mastering new situations” [6].	Activities that yield positive results may be more likely to be fun.

Performance orientation [6]	“A desire to demonstrate competence and be positively evaluated by others, which can be further divided into prove and avoid dimensions. A prove goal orientation focuses on demonstrating competence, whereas an avoid goal orientation focuses on not revealing incompetence and avoiding negative ability judgments from others” [6].	People may be more likely to have fun with activities that demonstrate competence and mitigate negative judgments. Conversely, activities that demonstrate incompetence or evoke negative judgments may be less likely to be viewed as fun.

Personality systems interaction theory [34]	“Personality systems interaction theory postulates 2 executive control modes in volitional action: self-control and self-regulation (self-motivation). Self-control should deplete energy, whereas self-motivation should maintain energy and result in invigoration” [34].	Fun activities are proposed to engage the self-motivation mode, allowing people to immerse themselves in the fun experience.

Playfulness [28]	A predisposition to define and engage in activities in a nonserious or fanciful manner to increase enjoyment. May be more process oriented than work that is results oriented, also intrinsically motivated and more spontaneous.	Playfulness is closely associated with fun, especially with children, and may help inform how fun can be promoted.

Positive psychology [28]	The study of what makes people happy and fulfilled. Emphasizes concepts such as resilience, optimism, joy, flow, and creativity.	Fun is a positive affect state in the domain of positive psychology.

Psychological safety [6]	“A climate of psychological safety is characterized by trust, respect, concern for others, and confidence in members’ abilities” [6]	People may be more likely to have fun in an environment of psychological safety.

Returnance [12]	“The desire to do an enjoyable activity again and again” [12].	Fun activities may demonstrate high returnance.

Self-determination theory [6,57]	“When the 3 needs of competence, autonomy, and relatedness are satisfied, intrinsic motivation is increased. In a training context, motivation to learn can be enhanced by the degree to which the training design and delivery helps trainees meet these three needs” [6].	When activities are fun, people are more likely to find intrinsic motivation, driven by personal interest rather than external rewards.

Social boundaries [37,62]	“The outer limit of what one sees as allowable, understandable or feasible” [62].	Social boundaries may mediate what is considered fun in different social groups.

Three stages of interest [36,63]	Triggered situational interest is sparked by stimuli that attract attention. Stabilized situational interest is marked by active engagement and intrinsic motivation. Individual interest is characterized by enduring commitment and personal investment.	Fun activities may trigger interest and maintain interest throughout all 3 phases.


### Outcomes of fun

Articles listed numerous potential beneficial or negative outcomes of fun. We did not appraise the strength of evidence for these outcomes or systematically differentiate between empirical findings and conceptual associations. When fun was measured, its presence was typically assessed in 1 of 3 ways: 1) self-reported fun (persons state whether they are having fun or rate, based on a scale, how much fun they are having); 2) observed fun (an external observer perceives a person to be experiencing fun); and 3) surrogate-measured fun (investigators measure a feature or activity assumed to be indicative of fun).

We grouped the purported beneficial outcomes of fun into 4 categories: personal well-being, social interaction, motivation, and performance. For *personal well-being*, articles explored whether the presence of fun can help reduce negative emotions (e.g., burnout [20], anxiety [38], emotional exhaustion [27], and stress [26]), improve positive emotions (positive mental attitude [39], enthusiasm [17], pride [16], and job satisfaction [14]), and improve health generally (enhanced quality of life [25] and well-being [21]). Regarding *social interaction*, the articles discussed how fun fosters collaboration [40], group cohesion [16], social connection [21], open communication [8], and a safe, inclusive environment [37]. Fun was reported to boost *motivation* and desire to learn by driving exploration [21], a sense of meaningfulness [8], curiosity [41], and enthusiasm [26]. From a *performance* perspective, the articles reported on how fun contributes to learning [8,21,42], improves employee retention [37], enhances creativity and concentration [21], and ultimately increases work productivity [25,26].

The potential negative outcomes of fun were noted less frequently. Fun may have negative connotations in professional settings. It may be considered flippant, escapist, silly, unprofessional, and trivial [13,14,43]. Articles described how the freedom inherent in many fun activities may create a chaotic atmosphere, leading to unproductive work [6,15]. When fun is coercive or introduced in inappropriate contexts, it risks becoming inauthentic and condescending, causing cynicism and misery for those forced to participate in the associated activities [8,38,44,45]. For a full list of outcomes, see Appendix D.

### Factors influencing fun

The articles explored 2 broad domains of fun-related factors: the features of activities that made them more likely to *be* fun and the characteristics of people that made them more likely to *have* fun. Although few articles addressed the latter, 6 personal characteristics were identified as conceptually linked with an increased tendency to have fun: younger age [6,30,46,47,48], emotional stability [6,45], being female [45], openness to experience [44], extroversion [44], and agreeableness [44].

In contrast, we identified more than 120 features of activities that may shape how fun they seem to participants. These features may either enable (e.g., interactivity [23], surprise [28], cooperation [22]) or inhibit (e.g., boredom [49], punishment [40], loneliness [28]) the fun of an activity. We grouped these features into 7 higher-order categories: safety, novelty, autonomy, playfulness, purpose, immersion, and social connection (Table 3, Appendix E). Each category includes features considered positive (enablers) or negative (inhibitors) as they relate to fun. No single feature was universally present, and the relevance of each feature varied by situation and person. A complex interplay of enablers and inhibitors appeared to exist simultaneously in all activities regardless of whether they are considered fun, leading one author to recommend that “different dimensions of fun should be examined separately as they may not be of equal importance” [45].

**Table 3:** Features of Fun Activities Identified in a Scoping Review of Fun in Education.


CLASSIFICATION LABEL	DEFINITION AND EXPLANATION	FUN ENABLERS^a^	FUN INHIBITORS^a^

1. Safety	Safety refers to environments that minimize physical and psychological danger, threat, stress, and pain [6,24]. An overwhelming sense of fear or danger is unlikely to be present during fun activities [13,40].	Peacefulness Pleasantness Psychological safety Relaxation Respect of boundaries Safe environment	Hostility Danger Pain Punishment Stress Threat

2. Novelty	Novelty is a feature of activities that are fresh or spontaneous, often involving surprise or adventure. Activities may be less fun when they become repetitive, mundane, or boring [14,22,27,28].	Adventure Curiosity Freshness Spontaneity Surprise	Mundanity Repetitiveness Tedium Boredom

3. Autonomy	Autonomy signifies freedom, agency, and choice, highlighting that individual decisions can influence outcome [22,38,41,46]. Activities that present a lack of choice or involve mandatory participation may be less fun [13,22].	Sense of control Freedom Choice Flexibility Ownership Voluntary participation	Compulsion Obligation Constraint

4. Playfulness	Playfulness indicates that the activity is humorous, informal, imaginative, or fantastic and may involve laughter, wackiness, frivolity, and storytelling [8,11,13,24,35,37]. Activities may not be fun if they are too serious.	Creativity Fantasy Humor Imagination Storytelling Frivolity	Strictness Excessive seriousness

5. Purpose	Purpose refers to the sense that activities are more fun if people have a reason to participate [8,15,21]. Activities seen as pointless or a waste of time are less likely to be considered fun [13,52].	Accomplishment Rewards Extrinsic positive outcome High personal value Intrinsic fulfillment Relevance	Pointlessness Excessive duration Focus on material gain Hassle Incompetence Time wasting

6. Immersion	Immersion refers to activities that are engaging, interesting, experiential, and stimulating [6,14,22,35,36]. Activities are considered less fun if they are dull, uninteresting, or rushed [24,30,43].	Active participation Attention Appropriate challenge Engagement Interest Physicality	Apathy Distraction Dullness Limited involvement Passivity Hurry

7. Social connection	Social connection is present when activities are experienced with others and there is a sense of belonging, connection, cooperation, or bonding [14,22,26,33]. Activities are less likely to be fun if they are associated with a negative social experience or isolation [8,13,22,29,30,35,38].	Bonding Cooperation Friendship Loss of social barriers Social events Trust	Condescension Exclusion Isolation Loneliness Pretentiousness Unfriendliness


^a^ For a complete list of features identified in the reviewed studies, see Appendix E.

*Safety* enablers included psychological or physical safety [6,24]. Safety inhibitors included danger, threat, stress, or pain. Although a certain amount of perceived danger may exist in fun activities (e.g., thrilling or suspenseful), an overwhelming sense of fear or danger was seen as a safety inhibitor [13,40]. The same situation could, at times, work both ways; for example, many people find skydiving to be fun and exhilarating, whereas others may find it terrifying and not fun at all.

*Novelty* refers to the fresh or spontaneous nature of fun activities. Novelty enablers may feature an element of surprise or adventure. Novelty inhibitors are seen when activities become repetitive, mundane, or boring [14,22,28]. In fact, boredom stands out as arguably the feature most antithetical to fun [22,27]. Although activities may exist that are neither fun nor boring, conceiving of an activity that is simultaneously both boring and fun would be challenging, if not impossible.

*Autonomy* has a paramount role in fun activities, as described in various articles. Fun activities are enabled by a sense of control and agency [38]. Fun activities typically allow participants to influence how the activity unfolds and, in many cases, its outcome. Other fun enablers include freedom, ownership, and interactivity [22,38,41,46]. Conversely, a lack of choice or mandatory participation were viewed as autonomy inhibitors [13,22].

*Playfulness* enablers include humor, informality, imagination, fantasy [24,35,37], laughter, wackiness, frivolity, and storytelling [8,11,13]. Playfulness is so strongly associated with fun that some authors argue that it is a requisite component for defining and recognizing fun [25,28,47]. For children, these two terms are often used interchangeably, as seen in the expression “fun and games” [50]. Activities may become less fun if they are too serious, and strictness was seen as a fun inhibitor.

*Purpose* refers to the notion that activities are more fun when people have a reason to participate [21]. Enablers include activities holding an increased expected task value [51], which may manifest in benefits such as praise [8], social capital [43], learning [33], developmental opportunities [21], or a tangible reward [15]. An activity with a highly relevant purpose may be perceived as more fun [21,35]. Conversely, purpose inhibitors include the perception that activities are pointless or a waste of time [13,52].

*Immersion* enablers include features such as engaging, interesting, and experiential [6,14,36]. Immersive activities often encourage active participation and provide sensory stimulation [22,35]. They strike the delicate balance between too much challenge and too little challenge, promoting a state of flow [12,28]. In contrast, immersion inhibitors include features such as dull, uninteresting, or rushed [24,30,43]. When people approach activities with apathy or face excessive distraction, their likelihood of experiencing fun diminishes [14,40].

*Social connection* enablers occur when activities are shared with others, creating a sense of belonging. As highlighted in one article, “Fun is more fun when others are involved” [29]. Fun activities, such as social events and teambuilding exercises, can break down social barriers and foster connections, cooperation, and bonding [14,22,26,33]. Conversely, social connection inhibitors are experienced in negative social interactions including condescension, unfriendliness, aggressive competition, exclusion, isolation, loneliness, and marked power differentials [8,13,22,29,30,35,38].

### Definition of fun

Our scoping review did not reveal a consistent definition of fun that was agreed upon across the articles. Of the 40 reviewed articles, 11 (27.5%) did not define fun. Of the remaining 29 articles, 16 (55%) included multiple definitions. We identified 58 discrete instances of fun being defined (including definitions repeated in multiple articles): 23 (40%) seemed novel to that article and 35 (60%) were quoted from previous sources. Only 9 of the 23 novel definitions (39%) cited prior work (i.e., building on existing theories or prior definitions of fun); the others appeared to be created from the authors’ personal ideas without referencing earlier work. Appendix B contains a full list of definitions. Common themes among the definitions included:

1. Positive valence emotional state: Nearly all definitions linked fun with positive emotions such as enjoyment, pleasure, and amusement [6,8,11,12,21,23,25,26,30,37,40,43,47,53].
2. Engagement and participation: Fun was frequently tied to active involvement or participation, whether in work, play, learning, or social activities [6,11,21,23,25,26,28,37,40,51].
3. Light-heartedness: A playful or humorous nature was commonly described, suggesting that fun often includes elements of creativity, laughter, and frivolity [8,11,13,24,25,28,35,37,47,50].
4. Motivation: Fun was often associated with activities that people engaged in for their inherent satisfaction, often described in terms of immersion or flow [6,23,26,28,29,43].

Three definitions were quoted repeatedly (more than 5 times each):

1. Fluegge (2008): “Fun at work involves any social, interpersonal, or task activities at work of a playful or humorous nature, which provide an individual with amusement, enjoyment, or pleasure” [25].
2. Ford and colleagues (2003): “A fun work environment intentionally encourages, initiates, and supports a variety of enjoyable and pleasurable activities that positively impact the attitude and productivity of individuals and groups” [43].
3. Peluchette and Karl (2005): “Experienced fun is the extent to which a person perceives the existence of fun in the workplace” [54].

Each of these highly-cited definitions refers explicitly to workplace settings, and applying these to other contexts such as traditional education requires both rephrasing and conceptual adjustment. An additional limitation of Peluchette and Karl’s [54] definition lies in its circularity—it uses the existence of fun to define the experience of fun. Many other definitions associate fun with the closely related concept of *enjoyment*. Although enjoyment represents a general feeling of pleasure, fun implies a more active and engaging experience. Notably, none of the cited definitions fully incorporates all the key themes identified in our synthesis of reviewed articles.

Integrating the salient findings from all articles included in our review, we synthesized a working definition of fun, which we propose could generalize across most contexts, including education: “Fun is a positive emotional experience arising from active engagement in enjoyable activities or experiences, often of a playful or amusing nature, that naturally fosters a desire to sustain or revisit the activity.”

## Discussion

Although established educational and psychological frameworks explain many of the factors underlying learner engagement and motivation, learners and educators continue to describe educational experiences using the term *fun* [14]. The present review synthesizes how this construct has been conceptualized across diverse literatures, providing a clearer conceptual foundation for future research. Our scoping review highlighted several important features of fun in education. Fun was consistently linked to positive emotions that are often accompanied by active participation, increased motivation, and a desire to continue or repeat enjoyable activities. The associated conceptual frameworks highlight the cognitive, emotional, and situational features through which fun has been conceptualized. We grouped the features of fun activities into 7 categories: safety, novelty, autonomy, playfulness, purpose, immersion, and social connection. We found that most definitions of fun were vague, poorly grounded, circular, or altogether absent, so we synthesized key findings into a working definition of fun. A more consistent conceptual foundation may facilitate clearer communication, improve comparability across studies, and provide a starting point for future work on operationalization, measurement, and empirical validation.

### Implications

Our findings may have significant implications for all fields of education. Our summative conceptual synthesis combines findings from education [7,12,14,21,22,24,26,32,35,36,41,49,53], workplace studies [6,15,25,27,28,30,37,38,40,43,45,47,54,55], and cognitive psychology [8,12,29,34,36,41,46,51,53] to create a framework for guiding future research. It builds off a 2019 review by Tews and Noe [6], which helped solidify our understanding of the role of psychological engagement in fun learning. That review concluded with a call to understand “how fun influences learning in different types of training and what aspects of fun are responsible.” The current study provides a categorization of the factors that govern the way learners relate to activities through fun. This will allow for more purposeful investigation into fun and its effects, both positive and negative.

Our review identified more than 120 features of fun activities, organized into 7 categories and identified as either enablers or inhibitors. Though alternative categorizations could have been made by other researchers, we consider this framework to be particularly useful for educators and researchers alike. We note that not all features were supported by empirical evidence. However, it seems reasonable to use these enablers and inhibitors as a starting point in designing fun activities, but further research is needed to confirm their benefit.

Although several outcomes were associated with fun activities, the literature was characterized by considerable conceptual heterogeneity, with many outcomes examined in only a small number of studies and relatively little replication across investigations. This fragmentation limits the accumulation of evidence and highlights the need for future research using more consistent definitions, measures, and outcomes. Such refinement could lead to more meaningful investigation into situations where fun is not just a frivolous pursuit but a powerful tool for enhancing learning, motivation, and overall well-being.

Our findings revealed a total of 22 conceptual frameworks conceptually linked with fun such as flow [56] and self-determination theory [57]. Some of these frameworks address the features of fun activities, whereas others address characteristics of individuals and how they experience fun. Associating fun with existing conceptual frameworks offers a way to rapidly advance our understanding of its mechanisms and effects. Clarifying empirical relationships between these conceptual frameworks and fun represents an important direction for future research.

Our findings suggest that fun should be understood within the context of both characteristics of individual learners and the unique setting in which the activity occurs. The classifications and definitions outlined in this article offer a foundation for developing hypotheses to identify which features of fun activities are most influential in specific groups and environments. Although research on fun has largely focused on inhibitors and enablers of fun, little attention has been given to characteristics of learners that may enhance the experience of fun. This gap presents an important opportunity to investigate how individual learner characteristics can shape engagement through fun, as well as to explore how fun may enhance engagement without introducing distraction.

Most importantly, our analysis uncovered recurring key themes in defining fun, which informed a synthesized working definition of fun. This broadly applicable definition addresses gaps in prior work by integrating themes recurring across diverse contexts, including workplace and educational settings. In particular, this working definition may prove valuable in educational settings, including health professions education, where fun has rarely been studied as a tool for engagement. This definition, along with the other insights gained in the review, provides educators and researchers with a conceptual foundation for future investigation that may inform instructional design.

### Limitations and strengths

Our synthesis may reflect biases in the reviewed literature, including publication bias and potentially overstated results. We did not search the Education Resources Information Center (ERIC) or Web of Science, and therefore some relevant educational studies may not have been identified. We intentionally included nonempirical articles and studies of varying quality to provide a comprehensive overview of the current state of the field and to delineate areas for further research into fun in education. This approach broadens the scope of our findings but limits our ability to draw conclusions about empirical support for the associations and outcomes related to fun. Many of the reviewed articles relied heavily on conceptual associations rather than on robust empirical data. Also, in alignment with the methods and purposes of a scoping review, we did not appraise quality or extract detailed results for original research studies. Finally, many included studies were conducted in workplace settings, potentially limiting generalizability directly to formal educational environments.

Strengths of this review include the purposeful and iteratively improved search strategy, extraction in duplicate of key article features, and linkage of fun with existing concepts. Our positionality as medical educators from a diverse interdisciplinary team provided a robust interpretive framework for adapting insights from other fields to HPE.

## Conclusion

These findings suggest a theory-informed conceptual framework of fun in learning that may provide a foundation for guiding future research into fun as an educational tool. This synthesized working definition of fun addresses gaps in prior research by incorporating recurring themes that are adaptable across diverse contexts, including health professions education.

## Additional File

The additional file for this article can be found as follows:

10.5334/pme.3030.s1 Appendices. Appendix A to E.

## Data Availability

Supplementary materials supporting this review are provided in Appendices A–E and include the complete search strategies, characteristics and findings of included articles, attributes and outcomes associated with fun, and features identified as enablers or inhibitors of fun.

## References

[1] Kassab SE, Al-Eraky M, El-Sayed W, et al. Measurement of student engagement in health professions education: a review of literature. BMC Med Educ. 2023;23(1):354. DOI: 10.1186/s12909-023-04344-8 PMC10199536 37210491

[2] Bakker AB, Mostert K. Study Demands–Resources Theory: Understanding Student Well-Being in Higher Education. Educ Psychol Rev. 2024;36(3):92. DOI: 10.1007/s10648-024-09940-8

[3] de Bruin ABH, Biwer F, Hui L, et al. Worth the Effort: the Start and Stick to Desirable Difficulties (S2D2) Framework. Educ Psychol Rev. 2023;35(2):41. DOI: 10.1007/s10648-023-09766-w

[4] Evans P, Vansteenkiste M, Parker P, et al. Cognitive Load Theory and Its Relationships with Motivation: a Self-Determination Theory Perspective. Educ Psychol Rev. 2024;36(1):7. DOI: 10.1007/s10648-023-09841-2

[5] Grund A, Fries S, Nückles M, et al. When is Learning “Effortful”? Scrutinizing the Concept of Mental Effort in Cognitively Oriented Research from a Motivational Perspective. Educ Psychol Rev. 2024;36(1):11. DOI: 10.1007/s10648-024-09852-7

[6] Tews MJ, Noe RA. Does training have to be fun? A review and conceptual model of the role of fun in workplace training. Hum Resour Manag Rev. 2019;29(2):226–238. DOI: 10.1016/j.hrmr.2017.11.003

[7] Tews MJ, Michel JW, Noe RA. Does fun promote learning? The relationship between fun in the workplace and informal learning. J Vocat Behav. 2017;98:46–55. DOI: 10.1016/j.jvb.2016.09.006

[8] Tews MJ, Jackson K, Ramsay C, et al. Fun in the college classroom: examining its nature and relationship with student engagement. Coll Teach. 2015a;63(1):16–26. DOI: 10.1080/87567555.2014.972318

[9] Eva KW, Cummings BA. With great power comes great responsibility: How narrow conceptions of validity in high-stakes testing undermine competence. Med Educ. 2026. DOI: 10.1111/medu.70252 42324172

[10] D’Eon MF. The overcrowded curriculum is alarming. Can Med Educ J. 2023;14(4):1–5. DOI: 10.36834/cmej.78084 PMC10500406 37719410

[11] Tasci ADA, Ko YJ. A FUN-SCALE for understanding the hedonic value of a product: the destination context. J Travel Tour Mark. 2016;33(2):162–183. DOI: 10.1080/10548408.2015.1038421

[12] Tisza G, Markopoulos P. FunQ: measuring the fun experience of a learning activity with adolescents. Curr Psychol. 2023;42:1936–1956. DOI: 10.1007/s12144-021-01484-2

[13] Karl K, Peluchette J, Hall-Indiana L, et al. Attitudes toward workplace fun: a three sector comparison. J Leadersh Organ Stud. 2005;12(2):1–17. DOI: 10.1177/107179190501200201

[14] Lucardie D. The impact of fun and enjoyment on adult’s learning. Procedia Soc Behav Sci. 2014;142:439–446. DOI: 10.1016/j.sbspro.2014.07.696

[15] Karl KA, Peluchette JV. Does workplace fun buffer the impact of emotional exhaustion on job dissatisfaction? A study of health care workers. J Behav Appl Manag. 2006;7(2):128–142. DOI: 10.21818/001c.16553

[16] Arksey H, O’Malley L. Scoping studies: towards a methodological framework. Int J Social Res Methodol. 2005;8(1):19–32. DOI: 10.1080/1364557032000119616

[17] Levac D, Colquhoun H, O’Brien KK. Scoping studies: advancing the methodology. Implement Sci. 2010;5(69). DOI: 10.1186/1748-5908-5-69 PMC2954944 20854677

[18] Cooper S, Cant R, Kelly M, et al. An Evidence-Based Checklist for Improving Scoping Review Quality. Clin Nurs Res. 2021;30(3):230–240. DOI: 10.1177/1054773819846024 31088144

[19] Covidence systematic review software. Veritas Health Innovation, Melbourne, Australia. Available from: https://www.covidence.org/.

[20] Russell JA. Core affect and the psychological construction of emotion. Psychol Rev. 2003;110(1):145–172. DOI: 10.1037/0033-295x.110.1.145 12529060

[21] Strean WB, Holt NL. Coaches’, athletes’, and parents’ perceptions of fun in youth sports: Assumptions about learning and implications for practice. Avante. 2000;6(3):83–98.

[22] Dismore H, Bailey R. Fun and enjoyment in physical education: young people’s attitudes. Res Pap Educ. 2011;26(4):499–516. DOI: 10.1080/02671522.2010.484866

[23] Vieira LC, Corrêa da Silva FS. Assessment of fun in interactive systems: a survey. Cogn Syst Res. 2017;41:130–143. DOI: 10.1016/j.cogsys.2016.09.007

[24] Elton-Chalcraft S, Mills K. Measuring challenge, fun and sterility on a ‘phunometre’ scale: evaluating creative teaching and learning with children and their student teachers in the primary school. Education 3–13. 2013;43(5):482–497. DOI: 10.1080/03004279.2013.822904

[25] Fluegge ER. Who put the fun in functional? Fun at work and its effects on job performance. Gainesville, FL: University of Florida; 2008.

[26] Sim G, MacFarlane S, Read J. All work and no play: measuring fun, usability, and learning in software for children. Comput Educ. 2006;46(3):235–248. DOI: 10.1016/j.compedu.2005.11.021

[27] Bolton SC, Houlihan M. Are we having fun yet? A consideration of workplace fun and engagement [Editorial]. Employee Relat. 2009;31(6):556–568. DOI: 10.1108/01425450910991721

[28] McDowell T. Fun at work: scale development, confirmatory factor analysis, and links to organizational outcomes. San Diego (CA): Alliant International University; 2004.

[29] Reis HT, O’Keefe SD, Lane RD. Fun is more fun when others are involved. J Posit Psychol. 2017;12(6):547–557. DOI: 10.1080/17439760.2016.1221123 PMC5597001 28919919

[30] Tews MJ, Michel JW, Bartlett A. The fundamental role of workplace fun in applicant attraction. J Leadersh Organ Stud. 2012;19(1):105–114. DOI: 10.1177/1548051811431828

[31] Bisson C, Luckner J. Fun in learning: the pedagogical role of fun in adventure education. J Exp Educ. 1996;19(2):108–112. DOI: 10.1177/105382599601900208

[32] Fu F-L, Su R-C, Yu S-C. EGameFlow: a scale to measure learners’ enjoyment of e-learning games. Comput Educ. 2009;52(1):101–112. DOI: 10.1016/j.compedu.2008.07.004

[33] Visek AJ, Achrati SM, Mannix H, et al. The fun integration theory: toward sustaining children and adolescents sport participation. J Phys Act Health. 2015;12(3):424–433. DOI: 10.1123/jpah.2013-0180 PMC4201634 24770788

[34] Kazen M, Kuhl J, Leicht EM. When the going gets tough…: Self-motivation is associated with invigoration and fun. Psychol Res. 2015;79(6):1064–1076. DOI: 10.1007/s00426-014-0631-z 25433692

[35] Chu SL, Angello G, Saenz M, et al. Fun in making: understanding the experience of fun and learning through curriculum-based making in the elementary school classroom. Entertain Comput. 2017;18:31–40. DOI: 10.1016/j.entcom.2016.08.007

[36] Ainley M, Ainley J. Student engagement with science in early adolescence: the contribution of enjoyment to students’ continuing interest in learning about science. Contemp Educ Psychol. 2011;36(1):4–12. DOI: 10.1016/j.cedpsych.2010.08.001

[37] Plester B. Crossing the line: boundaries of workplace humour and fun. Employee Relat. 2009;31(6):584–599. DOI: 10.1108/01425450910991749

[38] Fleming P. Workers’ playtime? Boundaries and cynicism in a “Culture of Fun” program. J Appl Behav Sci. 2005;41(3):285–303. DOI: 10.1177/0021886305277033

[39] Tews MJ, Michel JW, Stafford K. Does fun pay? The impact of workplace fun on employee turnover and performance. Cornell Hosp Q. 2013;54(4):370–382. DOI: 10.1177/1938965513505355

[40] Pryor MG, Singleton LP, Taneja S, et al. Workplace fun and its correlates: a conceptual inquiry. Int J Manag. 2010;27(2):294–302.

[41] Chan SCH, Wan CLJ, Ko S. Interactivity, active collaborative learning, and learning performance: the moderating role of perceived fun by using personal response systems. Int J Manag Educ. 2019;17(1):94–102. DOI: 10.1016/j.ijme.2018.12.004

[42] Spitzer RL, Kroenke K, Williams JB. Validation and utility of a self-report version of PRIME-MD: the PHQ primary care study. Primary Care Evaluation of Mental Disorders. Patient Health Questionnaire. JAMA. 1999;282(18):1737–1744. DOI: 10.1001/jama.282.18.1737 10568646

[43] Ford RC, McLaughlin FS, Newstrom JW. Questions and answers about fun at work. Hum Resour Plan. 2003;26(4):18–33.

[44] Karl KA, Peluchette JV, Harland L. Is fun for everyone? Personality differences in healthcare providers’ attitudes toward fun. J Health Hum Serv Adm. 2007;29(4):409–447. DOI: 10.1177/107937390702900403 17571467

[45] Becker FW. The impact of fun in the workplace on experienced fun, work engagement, constituent attachment, and turnover among entry-level service employees. University Park, PA: Pennsylvania State University; 2012.

[46] Tews MJ, Michel J, Xu S, et al. Workplace fun matters … but what else? Employee Relat. 2015b;37(2):248–267. DOI: 10.1108/ER-10-2013-0152

[47] Lamm E, Meeks MD. Workplace fun: the moderating effects of generational differences. Employee Relat. 2009;31(6):613–631. DOI: 10.1108/01425450910991767

[48] Tews MJ, Michel JW, Allen DG. Fun and friends: the impact of workplace fun and constituent attachment on turnover in a hospitality context. Hum Relat. 2014;67(8):923–946. DOI: 10.1177/0018726713508143

[49] Baid H, Lambert N. Enjoyable learning: the role of humour, games, and fun activities in nursing and midwifery education. Nurse Educ Today. 2010;30(6):548–552. DOI: 10.1016/j.nedt.2009.11.007 20044181

[50] Yogman M, Garner A, Hutchinson J, et al. The Power of Play: A Pediatric Role in Enhancing Development in Young Children. Pediatrics. 2018;142(3). DOI: 10.1542/peds.2018-2058 30126932

[51] Read JC, MacFarlane SJ, Casey C. Endurability, engagement and expectations: measuring children’s fun. Proceedings of the International Workshop on Interaction Design and Children (IDC 2002); 2002; Eindhoven, The Netherlands.

[52] Karl KA, Harland LK, Peluchette JV, et al. Perceptions of service quality: what’s fun got to do with it? Health Mark Q. 2010;27(2):155–172. DOI: 10.1080/07359681003745097 20446139

[53] Hsbollah HM, Hassan H. Creating meaningful learning experiences with active, fun, and technology elements in the problem-based learning approach and its implications. Malaysian J Learn Instr. 2022;19(1):147–181. DOI: 10.32890/mjli2022.19.1.6

[54] Peluchette J, Karl KA. Attitudes toward incorporating fun into the health care workplace. Health Care Manag (Frederick). 2005;24(3):268–275. DOI: 10.1097/00126450-200507000-00011 16131937

[55] Karl K, Peluchette J. How does workplace fun impact employee perceptions of customer service quality? J Leadersh Organ Stud. 2006;13(2):2–13. DOI: 10.1177/10717919070130020201

[56] Csikszentmihalyi M. Flow: the psychology of optimal experience. New York, NY: Harper & Row; 1990.

[57] Ryan RM, Deci EL. Self-determination theory and the facilitation of intrinsic motivation, social development, and well-being. Am Psychol. 2000;55(1):68–78. DOI: 10.1037//0003-066x.55.1.68 11392867

[58] Fredricks JA, Blumenfeld PC, Paris AH. School engagement: potential of the concept, state of the evidence. Rev Educ Res. 2004;74(1):59–109. DOI: 10.3102/00346543074001059

[59] Kahn WA. Psychological Conditions of Personal Engagement and Disengagement at Work. Acad Manage J. 1990;33(4):692–724. DOI: 10.5465/256287

[60] Gamorna A, Nystrand M. Taking students seriously. In: Newmann FM, editor. Student engagement and achievement in American secondary schools. New York: Teachers College Press; 1992. pp. 40–61.

[61] Hidi S, Renninger KA. The four-phase model of interest development. Educ Psychol. 2006;41(2):111–127. DOI: 10.1207/s15326985ep4102_4

[62] Managing Boundaries in Organizations: Multiple Perspectives. In: Paulsen N, Hernes T, editors. London: Palgrave Macmillan UK; 2003.

[63] Krapp A. Interest and human development: an educational–psychological perspective. In: Smith L, Rogers C, Tomlinson P, editors. Dev Motiv, BJEP Monograph Series. Development and motivation: joint perspectives. Leicester: British Psychological Society; 2003. pp. 57–84. DOI: 10.53841/bpsmono.2003.cat529.6

