# Supplementary material for: Taking Fun Seriously: A Scoping Review of Frameworks, Outcomes, and Definitions of Fun in Education
Source: Perspect Med Educ. 2026 Sep 18;15(1):845–60. doi: 10.5334/pme.3030 (PMC13588216; doi:10.5334/pme.3030)
Supplement: Supplementary file 1 [file pme-15-1-3030-s1.pdf]

Appendix A. Search Strategy for a Scoping Review of Fun in Education, 2025.

Source	Strategy
**PubMed**	• MeSH terms: Education, Medical OR Students, Medical OR Teaching OR Learning OR Happiness OR Personal Satisfaction<br>• Keywords (ti,ab): fun OR enjoyment OR engagement
**Medline**	1. exp Students, Medical<br>2. exp Education, Medical<br>3. fun.mp.<br>4. fun.ti,ab.<br>5. 3 or 4<br>6. 1 or 2<br>7. 5 and 6<br>8. limit 7 to english language
**Scopus**	• ((TITLE-ABS "medical education") OR TITLE-ABS ("medical students")) AND (TITLE-ABS-KEY (fun)

The initial search was performed in May 2022. The final search was performed in April 2024.

Appendix B. Summary of Main Findings and Definitions of Fun Identified in a Scoping Review of Fun in Education, 2025.

First author, year	Main findings	Definition of fun
Ainley, 2011	Findings supported a relationship between predictors of embedded interest. Specifically, the personal value of science and science knowledge were predictors of enjoyment of science and subsequently predict interest in learning science. In the model, this was a predictive path to embedded interest in science.	None
Baid, 2010	Identified themes within the literature: fun in educational contexts must have a meaningful connection to the topic being taught to provide a useful learning experience. Any humor used must be acceptable to the group, and the intention behind teaching strategies must be viewed by the group as friendly and positive. Additionally, educational technologies had the potential for more interaction and fun but were limited by the teacher’s effectiveness and skills.	None

Becker, 2012	Turnover in entry-level service employees positively correlated with nonwork–related fun activities and manager support for fun. Turnover negatively correlated with celebrations at work. No significant correlations were found between turnover and socializing with coworkers. Experienced fun, work engagement, and constituent attachment did not mediate facets of fun and employee turnover.	Same as definition from Peluchette (Peluchette & Karl, 2005)
Bolton, 2009	Suggested that research should focus on evaluation motivations, processes, and outcomes of organized fun at work. Also encouraged research into employees' reactions to organized fun at work in the dimensions of engagement, enjoyment, endurance, and escape.	None
Chan, 2019	Active collaborative learning mediated the relationship between interactivity and students' learning performance. High levels of experienced fun resulted in a stronger association between interactivity and	Fun experienced through personal response systems refers to the extent to which students find the use of personal response systems in

	students' learning performance.	the classroom fun and enjoyable.
Chu, 2017	In curriculum-based Maker activities, positive affect was associated with experimentation, novelty, wanting involvement, accomplishment, fantasy, wanting to get it done, and being absorbed in the activity. For social aspects, positive affect was associated with ordering others, sharing, answering, boasting about knowledge, chatter, instruction, and questioning. Negative affect was associated with wanting involvement, giving instruction, boasting about knowledge, and ordering others. Learning opportunities occurred when students asked questions and observed others.	None
Dismore, 2011	Fun was associated with positive attitudes and feelings toward physical education for primary and secondary school children. In primary school, fun was important for children to enjoy physical education. In secondary school, fun was related to students'	Definition 1: "A positive mood state associated with personal accomplishment and the ability to meet a challenge" Definition 2: "A trivialized factor or a hedonistic escapist

	having a challenge to overcome and not hedonic enjoyment.	notion of what enjoyment is"
Elton-Chalcraft, 2013	Effective and creative teaching and learning includes fun as a necessary component. As a construct, fun was distinct from appropriate challenges. Other necessary factors for effective and creative teaching and learning included achievement through intrinsic motivation, willingness to take risks and learn from mistakes, children's ownership of learning, and the teacher's role as facilitator.	Exciting and enjoyably challenging activity when there is pride in achievement after an initial struggle
Fleming, 2005	In some organizations, managed fun involved the blurring of work and nonwork boundaries, and this appeared to be partially associated with cynicism for some employees. Factors associated with cynicism are condescension and inauthenticity, such as managed fun that is perceived as "silly and zany."	None
Fluegge, 2008	Fun at work had direct and indirect effects on job performance. Fun at work positively correlated with organizational citizenship behavior, task performance, creative	Definition 1: Fun at work involves any social, interpersonal, or task activities at work of a playful

	performance, positive affect, and work engagement. Fun at work consisted of socializing with coworkers, celebrations at work, personal freedoms, and global fun.	or humorous nature that provide an individual with amusement, enjoyment, or pleasure.<br>Definition 2: Same as definition 3 from McDowell (McDowell, 2004)<br>Definition 3: Same as definition 1 from Ford (Ford et al., 2003)
Ford, 2003	Human resource managers had positive views about fun at work because of their beliefs that fun has individual and organizational benefits. Human resource managers promoted fun through many methods, such as celebrations, social events, stress-relieving activities, humor, games, and friendly competitions.	Definition 1: A fun work environment intentionally encourages, initiates, and supports a variety of enjoyable and pleasurable activities that positively affect the attitude and productivity of individuals and groups.<br>Definition 2: Fun at work is a work environment that makes people smile.

Fu, 2009	The EGameFlow measure is an effective tool to evaluate the level of enjoyment of e-learning games with satisfactory validity and reliability. The measure includes 8 dimensions: immersion, social interaction, challenge, goal clarity, feedback, concentration, control, and knowledge improvement.	None
Hsbollah, 2022	Problem-based learning activities integrated with Active Fun Technology elements demonstrated meaningful learning experiences and development of soft skills and technical skills.	Definition 1: Laughter and joking, freedom and creativity, and playfulness<br>Definition 2: 1) The hard level of difficulty associated with the problem, 2) the high level of activity demanded by the nature of the learning, and 3) the transformative nature of learning in terms of attitudinal change
Karl, 2005	There were no differences in attitudes towards fun across employees in the public, nonprofit, and private sectors. Employees in all sectors	None

	reported that food-related activities were fun. Employees in the public sector were more likely to report activities as less fun compared with the other 2 sectors. Overall, attitudes toward fun positively correlated with the employee’s trust in their supervisor and trust in their coworkers.	
Karl, 2006	Health care workers reported positive attitudes toward workplace fun. Health care workers who had higher levels of fun at work had significantly less negative impact of emotion exhaustion on job satisfaction. Employees who had a more positive attitude toward fun were more likely to experience fun in the workplace, have higher job satisfaction, and have less emotional exhaustion.	Same as definition from Peluchette (Peluchette & Karl, 2005)
Karl, 2007	The Big Five personality dimensions that positively correlated with attitudes toward fun were extraversion and agreeableness. Extraversion and emotional stability positively correlated with the amount of fun experienced. Employees who reported higher	Same as definition from Peluchette (Peluchette & Karl, 2005)

	levels of workplace fun had significantly lower emotional exhaustion and emotional dissonance and higher job satisfaction.	
Karl, 2010	Observing medical staff having fun in the workplace had no effect on customers' perceptions of service quality and behavioral intentions. However, the amount of time a customer waits may affect their perception of fun in the workplace.	Same as 2 definitions from Ford (Ford et al., 2003)
Karl, 2006	Employees who experienced workplace fun had greater job satisfaction, and this relationship was stronger for those who put a high value on workplace fun. Employees with adequate job satisfaction believed that their organization provided reliable, responsive, and empathetic customer service.	Same as 2 definitions from Ford (Ford et al., 2003)
Kazen, 2015	Results supported the personality systems interaction theory, which theorizes that there are 2 executive control modes in volitional action: self-control and self-regulation. During resource-demanding tasks, energy was depleted in the self-control group and the	None

	group had less fun, whereas the self-motivation group was invigorated and had more fun.	
Lamm, 2009	Generational membership moderated the relationship between workplace fun and job satisfaction and task performance; this relationship was strongest for millennials. Attitudes of boomers indicated a positive or nonsignificant relationship between workplace fun and job satisfaction. Organizational citizenship behavior was not moderated by generational membership.	Definition 1: Fun at work is taking work but not necessarily yourself seriously.<br>Definition 2: “When work and play are successfully intermixed to create a calm, stress-free, and happy environment”<br>Definition 3: Same as definition 1 from Ford (Ford et al., 2003)<br>Definition 4: Same as definition 3 from McDowell (McDowell, 2004)<br>Definition 5: Same as definition 1 from Fluegge (Fluegge, 2008)<br>Definition 6: Playful social,

		interpersonal, recreational, or task activities intended to provide amusement, enjoyment, or pleasure
Lucardie, 2014	Adult learners identified specific elements of experiencing fun in learning: activities, process established by teachers, interacting with others, humor, achievement, emotions and well-being, and personal benefits. Adult learners and teachers reported that fun and enjoyment are important to the learning experience, as they help motivate learners, enhance concentration, and improve learning knowledge and skills.	None
McDowell, 2004	A strong positive correlation was identified between a fun climate, job satisfaction, and an employee's emotional attachment to, identification with, and involvement in the organization. Fun climate factors included socializing with coworkers, celebrating at work, and global workplace fun.	Definition 1: A fun climate is "an organizational climate that supports fun at work, including management-supportive fun, socializing with coworkers, celebrating at work, and personal freedom at work."

		Definition 2: A fun person has an "individual disposition toward experiencing fun, including the tendency to be spontaneous, expressive, playful, and amused."
		Definition 3: Fun at work is "engaging in activities not specifically related to the job that are enjoyable, amusing, or playful."
Peluchette, 2005	Participants expressed positive attitudes regarding the appropriateness, salience, and consequences of having fun at work. Higher levels of workplace fun were significant for positive correlation with higher job satisfaction. Persons with more positive attitudes toward fun were more likely to experience fun in the workplace and have higher job satisfaction.	Experienced fun is the extent to which a person perceives the existence of fun in the workplace.
Plester, 2009	The organizational structure had a strong influence on boundary creation, such that	"Fun workplaces support pleasurable, enjoyable

	formal companies had narrower boundaries on humor and fun in the workplace, which constrained humor activities. In an informal company with wider boundaries, more unrestrained workplace humor and activities occurred.	activities and may include humorous events that promote laughter and/or pleasure."
Pryor, 2010	Workplace fun was experienced along a continuum of less fun to more fun. Organizational leaders and their employees can increase the potential for workplace fun through personal attitudes and actions. Workplace fun positively impacted safety, quality, productivity, and worker retention.	Definition 1: Fun is enjoyment and pleasure.<br>Definition 2: "Fun is the pleasure you experience while you are involved in some action such as doing something, seeing something, or even relaxing."
Read, 2002	A toolkit for measuring fun for children would include the Smileyometer or the Funometer (more useful for older children), an Again-Again table or a Fun-Sorter, together with observations (where appropriate) and remembrance metrics.	None

Reis, 2017	Fun was associated with high-activation and low-activation positive affect and was enhanced when experienced with others (especially friends). Social fun was associated with increases in high-activation but not low-activation positive affect, which suggests that social interaction emphasizes energizing affective experiences. Loneliness moderated effects, such that lonely people had a weaker boost from shared fun compared to solitary fun.	Fun-seeking is impulsivity and the desire for novelty and excitement.
Sim, 2006	Learning did not correlate with fun or usability when a software program was used to prepare children to take a government-issued science test. Observed fun and observed usability were correlated, and children of 7-8 years could differentiate between constructs to describe software quality. The Fun-Sorter appears to be an effective tool to evaluate products with children.	“Things are fun when they attract, capture, and hold our attention by provoking new or unusual emotions in contexts that typically arouse none, or arouse emotions not typically aroused in given context.”

Strean, 2000	Results suggested that coaches, parents, and children generally viewed drills as boring, whereas practice and competition games were fun. However, it was recognized that teaching skills through drills is necessary to be more successful in games. It was proposed that using the Teaching Games for Understanding theoretical framework may help coaches enhance children's fun in youth sport.	Definition 1: Fun in sports is to be totally involved, seeking pleasure from participation by oneself or with others. Definition 2: A general positive emotional state for young people Definition 3: The term fun ... signifies a positive emotional state that is a subset of enjoyment.
Tasci, 2016	The 12-item FUN-SCALE was developed through content analysis, data collection, exploratory factor analysis, and confirmatory factor analysis and comprises 4 factors: social vigor, psychological zest, emotional spark, and flow. Psychological zest was identified as the most important factor based on the grand mean, which may indicate that excitement, pleasure, and enjoyment experienced are important in the definition of fun.	Definition 1 (Dictionary.com): "Something that provides mirth or amusement" or "enjoyment or playfulness." Definition 2: "For the purposes of this study, fun is defined as a component of the hedonic value of an object resulting in desired sensory results for the consumer." Definition 3: "Positive sensory

		qualities leading to other correlates felt and appreciated by a person psychologically, socially, and sociopsychologically.”
Tews, 2012	Workplace fun was a stronger predictor of applicant attraction than compensation and opportunities for advancement among collegiate job seekers. Specifically, fun coworker interactions and fun job responsibilities were stronger predictors of applicant attraction than formal fun activities.	Same as definition 1 from Fluegge (Fluegge, 2008)
Tews, 2013	Fun in the workplace had both beneficial and potentially negative effects on restaurant server employees. Fun activities boosted performance, and manager support for fun reduced employee turnover. However, manager support for fun adversely affected performance. There was no difference in outcomes based on age of employees.	Definition 1: Same as definition from Peluchette (Peluchette & Karl, 2005) Definition 2: Same as definition 1 from Fluegge (Fluegge, 2008)
Tews, 2014	Coworker socializing and manager support for fun were significantly related to employee	Definition 1: Same as definition 1 from Fluegge

	turnover. Constituent attachment mediated the relationship between the 3 forms of fun and turnover. Fun influenced retention by facilitating the development of meaningful work relationships.	(Fluegge, 2008)<br>Definition 2: Same as definition 1 from Ford (Ford et al., 2003)<br>Definition 3: Same as definition from Peluchette (Peluchette & Karl, 2005)
Tews, 2015a	The multistage scale development effort resulted in a 2-dimensional measure, including fun activities and fun delivery. Fun activities included a variety of hands-on exercises and ways to promote social involvement among students. Fun delivery is instructor-focused, including the use of humor, creative examples, and storytelling. Fun delivery, but not fun activities, was positively related to student engagement.	Activities and interactions of an enjoyable, entertaining, humorous, or playful nature within a learning context
Tews, 2015b	Fun job responsibilities were the most dominant predictor of embeddedness (the overall degree to which an individual is enmeshed in the organization). Perceived career opportunities, praise, and rewards also predicted	Definition 1: Same as definition 1 from Fluegge (Fluegge, 2008)<br>Definition 2: Same as definition from Peluchette

	embeddedness. Coworker socializing and manager support for fun had modest influences on embeddedness.	(Peluchette & Karl, 2005)
Tews, 2017	Fun activities significantly correlated with overall informal learning; however, manager support for fun did not correlate. A negative interaction was found for predicting self-learning between core self-evaluations and fun activities, suggesting that fun may not benefit everyone equally. Overall, fun was a worthwhile strategy to promote informal learning, but it is important to consider the personality of learners to ensure fun has the intended impact.	Same as definition 1 from Fluegge (Fluegge, 2008)
Tews, 2019	From the article: “First, compared to other areas of training research, far fewer studies have investigated how fun features of the training environment influence learning outcomes. Second, there are mixed findings on fun’s relationship with trainees’ experiences and training outcomes, although trainee characteristics and the combination of training	Definition 1: “Environmental factors or features of the work environment of social, playful, and amusing nature” Definition 2: “A global holistic evaluation of whether a workplace provides enjoyment, amusement, or

	methods used likely affect these relationships. Third, researchers have not adopted a common theoretical basis for understanding fun training design and the area lacks a conceptual framework to motivate future studies.”	pleasure”<br>Definition 3: Same as definition 1 from Fluegge (Fluegge, 2008)<br>Definition 4: Same as definition 1 from Ford (Ford et al., 2003)<br>Definition 5: “The experience of positive affect, a state of high energy, full concentration, and pleasurable engagement.”<br>Definition 6: Same as definition 2 from Pryor (Pryor et al., 2010)
Tisza, 2023	Developed the 18-item Fun Questionnaire (FunQ) to assess fun of children, which demonstrated adequate validity and reliability. The 6 dimensions (autonomy, challenge, delight, immersion, loss of social barriers, and stress) were identified as core components of fun based on a literature review.	An informal expression for pleasure, enjoyment, or entertainment and as behavior or activities that are not serious; games or jokes.

Vieira, 2017	This literature review evaluated existing methods to assess fun and its core elements of attention, flow, immersion, and emotion. The authors stated that fun assessments included nonautomated (eg, expert analysis, observation, self-report) and automated (eg, biometric sensors) aspects. They noted that immersion and emotion are main components of fun, so automated assessments should include these elements.	Definition 1: "The pleasure and excitement experienced when playing games or doing other enjoyable activities." Definition 2: "Fun can be defined by 2 essential dimensions: immersion, as the increasing level of involvement with the task, that subsumes all the sensory, cognitive and emotional systems, and the emotions experienced by performing the task, which can be considered pleasurable despite their general likeness in other contexts."
Visek, 2015	Developed the theoretical framework FUN MAPS, which provides evidence-based guidelines for the fun integration theory. FUN MAPS includes 4 fundamental tenets of fun in youth sport within 11 fun dimensions with 81	None

specific fun determinants. This framework can be used to maximize fun for youth to help promote an active and healthy lifestyle through sport.

Abbreviation: FunQ, Fun Questionnaire; FUN MAPS, Framework of Fun Using a Novel Mixed-Methods Assessment of Participants in Sport; N/A, not applicable.

Appendix C. Attributes of Fun as Noted in Each Article Included in a Scoping Review of Fun in Education, 2025.[a]

	Ainley 2011	Baid 2010	Becker 2012	Bolton 2009	Chan 2019	Chu 2017	Dismore 2011	2013	Fleming 2005	Fluegge 2008	Ford 2003	Fu 2009	Hsbollah 2022	Karl 2005	Karl 2006	Karl 2007	Karl 2010	Karl 2006	Kazen 2015	Lamm 2009	Lucardie 2014	McDowel 2004	Peluchette 2005	Plester 2009	Pryor 2010	Read 2002	Reis 2017	Sim 2006	Strean 2000	Tasci 2016	Tews 2012	Tews 2013	Tews 2014	Tews 2015a	Tews 2015b	Tews 2017	Tews 2019	Tisza 2021	Vieira 2017	Visek 2015
Both a process & an outcome													x																											
Can be high or low arousal																											x												x	
Contagious																	x																							
Contextual																																							x	x
Desirable														x		x							x					x	x											
Different from work					x				x		x																													
Difficult to measure																												x												
Diffuses anger and distress		x																																				x		
Distorted sense of time																																						x		
Exists on a continuum																									x															
Holds attention					x																																			
Immersive																																							x	
Important														x		x																								
Increased arousal																	x																							
Intrinsically fulfilling						x																																		
Intrinsically motivating								x				x							x			x					x													
Less negative affect																											x										x			
Natural for children																										x			x											
Negative states are associated with a lack of fun, including:																																								
• Anxiety/worry							x		x												x	x															x			
• Confusion				x		x															x																			
• Fear														x							x	x			x															
• Frustration						x		x																				x												
• Uncertainty								x																																
Not all fun is equal																																		x						
Not boring		x		x			x	x	x												x	x							x											
Positive affect										x	x						x					x					x									x	x	x		
Positive valence emotional state	x	x			x	x	x	x		x					x		x	x		x		x	x				x												x	
Positive states associated with fun:																																								

• Amusement										x																											x		x	
• Delight																																						x		
• Enjoyment	x	x			x		x	x	x	x		x	x	x				x		x	x			x	x		x		x	x	x						x	x	x	
• Entertainment											x																													
• Excitement						x		x					x																	x										
• Happiness	x																			x										x										
• Humor/Joking													x		x	x				x				x								x			x					
• Frivolity																								x																
• Leisure, recreation																														x										
• Play				x		x	x		x	x	x		x	x				x			x	x		x						x		x			x					
• Pleasure									x	x	x	x					x	x		x				x	x			x		x							x		x	
• Satisfaction																																							x	
Related to object or activity						x					x										x			x																
Relative (different people find different things fun; affected by experience/background)								x					x		x	x				x		x	x		x	x			x	x		x		x				x	x	
Situational													x			x														x								x		
Socially engaging										x																													x	
Subjective																									x					x									x	
Subversive				x																																				

[a]An x marked on the table identifies the studies the observation was extracted from.

Appendix D. Benefits and Harms Associated with Fun as Noted in Each Article Included in a Scoping Review of Fun in Education, 2025.[a]

	Ainley 2011	Baid 2010	Becker 2012	Bolton 2009	Chan 2019	Chu 2017	Dismore 2011	2013	Fleming 2005	Fluegge 2008	Ford 2003	Fu 2009	Hsbollah 2022	Karl 2005	Karl 2006	Karl 2007	Karl 2010	Karl 2006	Kazen 2015	Lamm 2009	Lucardie 2014	McDowel 2004	Peluchette 2005	Plester 2009	Pryor 2010	Read 2002	Reis 2017	Sim 2006	Strean 2000	Tasci 2016	Tews 2012	Tews 2013	Tews 2014	Tews 2015a	Tews 2015b	Tews 2017	Tews 2019	Tisza 2021	Vieira 2017	Visek 2015
**Potential benefits**																																								
**1. Personal wellbeing**																																								
Decreased burnout			x																	x		x					x				x		x							
Enthusiasm								x																																
Feeling grown up							x																																	
Improves health																						x																		
Improves marriage outcomes																											x													
Job satisfaction					x						x				x	x	x	x		x		x	x	x	x		x			x	x	x	x		x	x				
Less emotional exhaustion			x	x						x					x	x	x															x				x				
Lowers stress		x	x	x							x			x	x			x				x			x							x	x		x			x		
Morale											x				x										x					x	x	x	x							
Positive mental attitude																				x		x														x				
Quality of life														x																										
Reduces anxiety																																	x	x						
Relaxation																						x																		
Sense of pride							x																										x							
Wellbeing																					x	x	x		x															
**2. Social interaction**																																								
Attractive work environment			x								x			x																	x		x		x	x				
Collaboration																																						x		
Employee retention			x	x							x	x		x	x		x					x			x						x	x	x			x				
Group cohesion/identity			x	x			x				x											x										x				x				
Interaction																																							x	
Open communication																																				x				
Promotes participation		x	x	x	x								x		x						x																			x
… especially at work										x	x																													
Reduces power differential		x																																						
Social connection/relationship			x	x		x					x		x							x	x	x	x	x						x					x	x				x
**3. Motivation**																																								
Exploration																					x																			
Increases meaningfulness																																				x	x			
Interest																																					x			

Job commitment				x						x	x			x	x							x			x		x			x		x	x		x	x				
Motivation/drive/desire		x		x		x		x		x	x	x	x	x	x	x	x				x		x						x							x	x	x	x
Satisfies curiosity																																						x	
**4. Performance**																																								
Concentration																					x																			
Creativity/innovation			x	x					x	x	x									x	x	x			x					x	x	x	x	x						
Customer satisfaction																	x	x					x		x								x		x					
Customer service			x						x						x	x							x								x									
Engagement			x	x						x	x													x						x				x	x	x	x	x		
Improves learning		x			x	x					x		x								x						x	x		x				x		x	x	x	x	
Increases energy																x	x																			x				
Reduces absenteeism											x		x		x		x								x						x									x
Task performance																			x						x											x	x			
Work productivity			x	x					x	x	x			x	x	x	x					x	x	x						x		x								
**Potential negatives**																																								
Blurring of work boundaries									x							x																								
Chaos														x	x					x			x														x			
Cognitive load																																					x			
Condescending									x																															
Cynicism									x	x				x		x		x					x									x			x					
Disharmony																								x																
Distracting											x							x			x				x		x									x	x			
Escapist							x				x																													
Exhausting/taxing								x							x	x																								
Expense											x																													
Glosses over real problems										x																														
Hedonistic							x	x																																
Inauthenticity			x																													x	x		x		x			
Increases errors											x							x																						
Irritating														x				x																						
Juvenile																																					x			
May be inappropriate context		x														x																								
… education		x					x																										x			x			
… healthcare														x		x						x																		
… work										x	x			x				x				x																		
May generate conflicts											x																													
May lose control											x																													
Misery				x							x					x																								
More work																							x																	
Offense																								x																
Opportunity cost																																					x			
Pressure to conform/coercive				x																				x			x													
Reduced time on task																																					x			

Reduces challenge		x																															x							
Silly, unprofessional											x			x	x			x				x																		
Triviality								x			x										x	x																		
Unsafe																									x															
Waste of time/too much time											x			x	x							x	x				x						x	x	x		x			

[a]An x marked on the table identifies the studies the observation was extracted from.

Appendix E. Characteristics of Activities Identified as Being Associated With an Increase in Fun or a Decrease in Fun as Noted in Each Article Included in a Scoping Review of Fun in Education, 2025.[a]

	Ainley 2011	Baid 2010	Becker 2012	Bolton 2009	Chan 2019	Chu 2017	Dismore 2011	2013	Fleming 2005	Fluegge 2008	Ford 2003	Fu 2009	Hsbollah 2022	Karl 2005	Karl 2006	Karl 2007	Karl 2010	Karl 2006	Kazen 2015	Lamm 2009	Lucardie 2014	McDowel 2004	Peluchette 2005	Plester 2009	Pryor 2010	Read 2002	Reis 2017	Sim 2006	Strean 2000	Tasci 2016	Tews 2012	Tews 2013	Tews 2014	Tews 2015a	Tews 2015b	Tews 2017	Tews 2019	Tisza 2021	Vieira 2017	Visek 2015
**1. Safety**																																								
**Increased fun**																																								
Peaceful						x																																		
Pleasant																				x																				
Psychological safety																																					x			
Relaxed																					x																x			
Respect of boundaries																								x																
Safe environment								x													x				x															
**Decreased fun**																																								
Aggression																						x																		
Bullying/harassment																									x															
Danger																								x																
Fatigue																						x																		
Fear											x			x								x			x															
Fragility						x																																		
Hostility														x											x															
Offensive humor																								x																
Pain																					x																			
Punishment																									x															
Stress						x			x						x						x									x								x		
Threat				x																					x												x			
Weakness																																					x			
**2. Novelty**																																								
**Increased fun**																																								
Adventure																														x										
Curiosity						x																								x									x	
Different learning modalities					x																																			
Fresh		x																																						
Novelty						x	x																																	
Spontaneous				x										x								x		x					x	x										
Surprise																						x								x										
**Decreased fun**																																								
Boredom		x		x			x	x	x												x	x							x											

Mundanity								x	x												x																			
Repetitiveness							x																																	
Tedium						x																																		
**3. Autonomy**																																								
**Increased fun**																																								
“Being myself”																														x										
Autonomy/sense of control				x		x	x	x	x			x																	x								x	x		
Fits with personal interests																															x									
Freedom/choice							x	x	x				x									x							x	x			x		x		x			
Giving orders						x																																		
Informal/casual									x												x																x			
Interactivity		x			x																				x															
Less bound by rules																					x																			
Ownership								x	x																															
Rebellious																														x										
Voluntary													x																									x		
**Decreased fun**																																								
Compulsion/mandatory			x	x					x					x		x																						x		
Lack of choice during activity							x		x																															
No sense of ownership						x			x																															
**4. Playfulness**																																								
**Increased fun**																																								
Creativity																									x					x				x						
Fantasy						x																								x									x	
Humor/laughter		x	x	x	x	x			x	x	x		x	x	x		x	x		x	x	x	x	x						x	x	x		x						
Imagination								x																																
Informality																								x																
Play/playfulness				x		x	x		x	x	x		x	x				x			x	x		x					x			x								
Storytelling																																		x						
Wackiness/frivolity/crazy				x		x			x									x												x										
**Decreased fun**																																								
Strictness							x							x																										
Too serious							x																																	
**5. Purpose**																																								
**Increased fun**																																								
Accomplish/achievement						x		x													x								x											
Awards/rewards/gifts			x												x		x	x					x						x											x
Competence																													x								x			x
Development opportunity			x				x				x	x																	x											x
Experimentation						x																																		
Extrinsic positive outcome							x																						x											x
Fun responsibilities																																			x		x			
High personal value	x																																							x
Intrinsically fulfilling						x	x														x																			

Praise																																		x						
Recognition			x							x	x												x									x								x
Relevance						x																							x											
**Decreased fun**																																								
Dragging on																					x																			
Focus on material gain																				x																				
Hassle														x																										
Incompetence																																				x			
Pointless/meaningless								x		x				x																										
Time wasting																x	x																							
**6. Immersion**																																								
**Increased fun**																																								
Absorbed in activity						x																																		
Active participation					x		x						x																x								x			
Activities			x				x		x							x	x	x			x											x		x		x	x			
Attention																																							x	
Challenge, not too much/little						x	x	x				x	x								x								x	x							x	x	x	
Engaging	x					x															x					x														
Experiential																																					x			
Hands-on																																			x		x			
Immersion												x																										x	x	
Integrated													x																											
Interesting	x	x					x														x																x			
Physical							x																																	
Sensual						x																																		
**Decreased fun**																																								
Apathy																									x															
Distraction						x															x																			
Dull/uninteresting								x																							x									
Feeling rushed											x																													
Limited involvement						x																																		
Passivity		x																																						
Too challenging							x													x																				
**7. Social connection**																																								
**Increased fun**																																								
Attention from others		x									x																												x	
… especially authority		x				x																							x											x
Authenticity (motives)									x																															
Bonding									x												x	x									x									
Cooperation							x		x																															
Decreased social barriers									x																															
Experienced with others			x				x					x									x	x					x		x	x			x	x	x					x
… especially friends/family																														x										
Friendship			x			x															x						x										x			x

Loss of social barriers/ suspension of social reality																																						x		
Positive emotions from others						x					x																										x			
Relationships/connection																					x									x	x			x						
Sense of belonging																																					x			
Sharing						x																																		
Social events			x				x			x	x											x										x				x	x			
Support from authority			x			x		x																x								x	x			x	x			
Teambuilding																																x				x				x
Trust			x							x				x																										
**Decreased fun**																																								
Aggressive competition							x																																	
Condescension									x																															
Disingenuous/fake									x																															
Exclusion						x																																		
High power differential																																		x						
Isolation																															x									
Loneliness																						x					x													
Pretentious										x																														
Unfriendliness			x											x											x															

[a]Characteristics were grouped into 7 categories: safety, novelty, autonomy, playfulness, purpose, immersion, and social connection. An x marked on the table identifies the studies the observation was extracted from.
